# Supplementary material for: Heteronuclear Dirhodium-Gold Anionic Complexes: Polymeric Chains and Discrete Units
Source: Polymers (Basel). 2020 Aug 19;12(9):1868. doi: 10.3390/polym12091868 (PMC7563758; doi:10.3390/polym12091868)
Supplement: Supplementary file 1 [file polymers-12-01868-s001.pdf]

Supplementary Materials

Heteronuclear Dirhodium-Gold Anionic Complexes: Polymeric Chains and Discrete Units

Estefania Fernandez-Bartolome[1], Paula Cruz[1], Laura Abad Galán[1], Miguel Cortijo[1], Patricia Delgado-Martínez[2], Rodrigo González-Prieto*[1], José L. Priego,[1], and Reyes Jiménez-Aparicio*[1]

[1] Departamento de Química Inorgánica, Facultad de Ciencias Químicas, Universidad Complutense de Madrid, Ciudad Universitaria, E-28040 Madrid, Spain.

[2] Unidad de Difracción de Rayos X. Centro de Asistencia a la Investigación de Técnicas Físicas y Químicas. Universidad Complutense de Madrid. Ciudad Universitaria. E-28040. Madrid. Spain

* Correspondence: rodgonza@ucm.es (R.G.-P.); reyesja@quim.ucm.es (R.J.-A.)

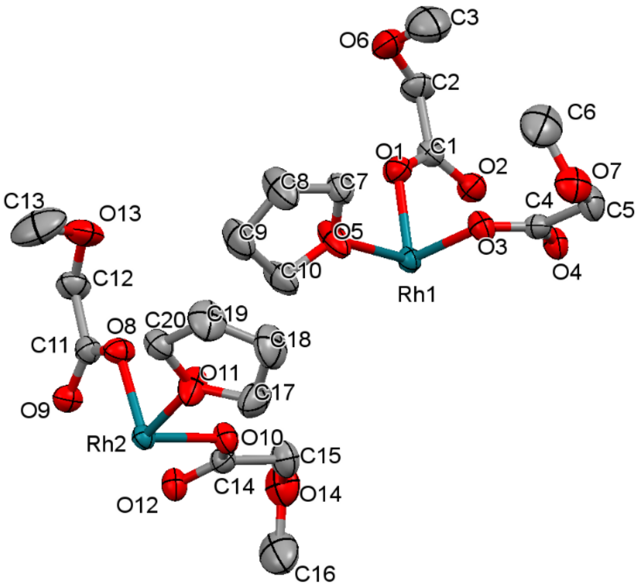

Figure S1. Representation of the asymmetric unit of [$Rh_2(\mu\text{-}O_2CCH_2OMe)_4(THF)_2$] (**1**) (50% probability ellipsoids). Rhodium: turquoise; oxygen: red; carbon: grey. Hydrogen atoms are omitted for clarity.

Table S1. Selected bond lengths [Å] and angles [°] for [$Rh_2(\mu\text{-}O_2CCH_2OMe)_4(THF)_2$] (**1**).

	**Bond length(Å)**		**Angle (º)**
Rh(1)-Rh(1)#1	2.3787(8)	O(1)-Rh(1)-Rh(1)#1	87.83(9)
Rh(2)-Rh(2)#2	2.3810(8)	O(3)-Rh(1)-Rh(1)#1	88.28(9)
O(1)-Rh(1)	2.031(3)	O(5)-Rh(1)-Rh(1)#1	178.43(12)
O(3)-Rh(1)	2.042(3)	O(8)-Rh(2)-Rh(2)#2	87.67(9)
O(5)-Rh(1)	2.256(3)	O(10)-Rh(2)-Rh(2)#2	88.24(9)
O(8)-Rh(2)	2.031(3)	O(11)-Rh(2)-Rh(2)#2	178.12(9)
O(10)-Rh(2)	2.034(3)		
O(11)-Rh(2)	2.258(3)		

Symmetry transformations used to generate equivalent atoms:

#1 -x+1,-y,-z+1 #2 -x,-y,-z

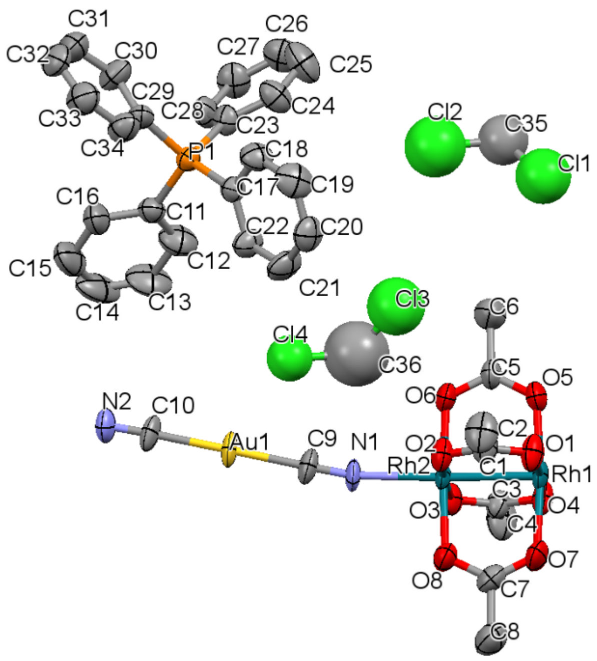

Figure S2. Representation of the asymmetric unit of $\{(PPh_4)[Rh_2(\mu\text{-}O_2CMe)_4Au(CN)_2]\cdot 2CH_2Cl_2\}_n$ (**3·2CH₂Cl₂**). (50% probability ellipsoids). Rhodium: turquoise; oxygen: red; carbon: grey; nitrogen: purple; gold: yellow; chlorine: green; phosphorus: orange. Hydrogen atoms are omitted for clarity.

Table S2. Selected bond lengths [Å] and angles [°] for $\{(PPh_4)[Rh_2(\mu\text{-}O_2CMe)_4Au(CN)_2]\cdot 2CH_2Cl_2\}_n$ (**3·2CH₂Cl₂**).

	**Bond length(Å)**		**Angle (º)**
Rh(1)-Rh(2)	2.3981(9)	O(1)-Rh(1)-Rh(2)	87.92(16)
O(1)-Rh(1)	2.037(6)	O(4)-Rh(1)-Rh(2)	88.23(15)
O(4)-Rh(1)	2.035(5)	O(5)-Rh(1)-Rh(2)	87.12(16)
O(5)-Rh(1)	2.031(6)	O(7)-Rh(1)-Rh(2)	87.59(17)
O(7)-Rh(1)	2.045(6)	O(2)-Rh(2)-Rh(1)	87.51(15)
Rh(1)-N(2)#2	2.221(7)	O(3)-Rh(2)-Rh(1)	87.53(15)
O(2)-Rh(2)	2.042(5)	O(6)-Rh(2)-Rh(1)	88.03(15)
O(3)-Rh(2)	2.039(6)	O(8)-Rh(2)-Rh(1)	88.43(16)
O(6)-Rh(2)	2.036(6)	N(1)-Rh(2)-Rh(1)	177.8(2)
O(8)-Rh(2)	2.033(6)	N(2)#2-Rh(1)-Rh(2)	179.0(2)
N(1)-Rh(2)	2.223(7)	C(9)-N(1)-Rh(2)	170.2(8)
		C(10)-N(2)-Rh(1)#1	169.8(8)

Symmetry transformations used to generate equivalent atoms:

#1 x-1,y,z   #2 x+1,y,z

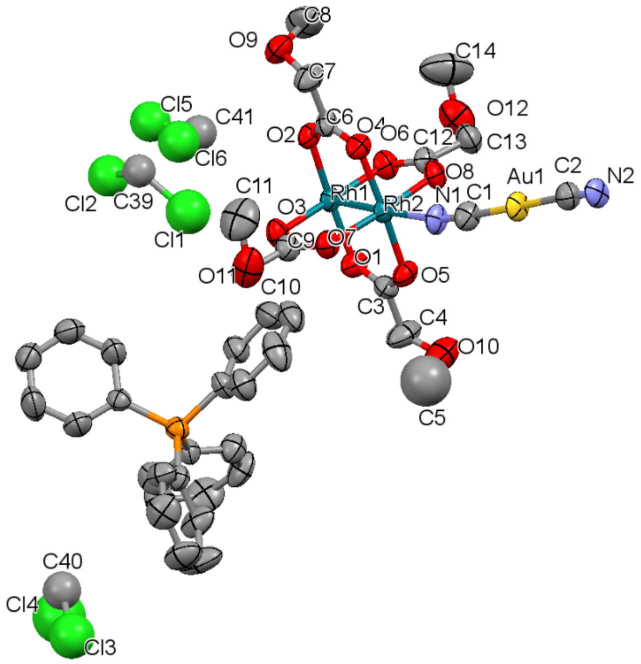

Figure S3. Representation of the asymmetric unit of $\{(PPh_4)[Rh_2(\mu\text{-}O_2CCH_2OMe)_4Au(CN)_2]\cdot 3CH_2Cl_2\}_n$ (**4·3CH$_2$Cl$_2$**) (50% probability ellipsoids). Rhodium: turquoise; oxygen: red; carbon: grey; nitrogen: purple; gold: yellow; chlorine: green; phosphorus: orange. The hydrogen atoms and the numbering of the $(PPh_4)^+$ cations (P1 and C15-C38) are omitted for clarity.

Table S3. Selected bond lengths [Å] and angles [°] for $\{(PPh_4)[Rh_2(\mu\text{-}O_2CCH_2OMe)_4Au(CN)_2]\cdot 3CH_2Cl_2\}_n$ (**4·3CH$_2$Cl$_2$**).

	**Bond length(Å)**		**Angle (º)**
Rh(1)-Rh(2)	2.4096(11)	O(1)-Rh(1)-Rh(2)	87.8(2)
O(1)-Rh(1)	2.033(8)	O(2)-Rh(1)-Rh(2)	87.98(19)
O(2)-Rh(1)	2.037(7)	O(3)-Rh(1)-Rh(2)	87.9(2)
O(3)-Rh(1)	2.038(8)	O(4)-Rh(1)-Rh(2)	87.6(2)
O(4)-Rh(1)	2.037(8)	O(6)-Rh(2)-Rh(1)	87.6(2)
N(2)-Rh(1)	2.187(9)	O(5)-Rh(2)-Rh(1)	88.0(2)
O(5)-Rh(2)	2.029(8)	O(7)-Rh(2)-Rh(1)	87.5(2)
O(6)-Rh(2)	2.042(7)	O(8)-Rh(2)-Rh(1)	87.6(2)
O(7)-Rh(2)	2.045(8)	N(1)-Rh(2)-Rh(1)	176.4(3)
O(8)-Rh(2)	2.038(8)	N(2)-Rh(1)-Rh(2)	177.2(3)
N(1)-Rh(2)	2.209(8)	C(1)-N(1)-Rh(2)	167.7(10)
		C(2)#2-N(2)-Rh(1)	170.4(10)

Symmetry transformations used to generate equivalent atoms:

#1 x-1,y,z   #2 x+1,y,z

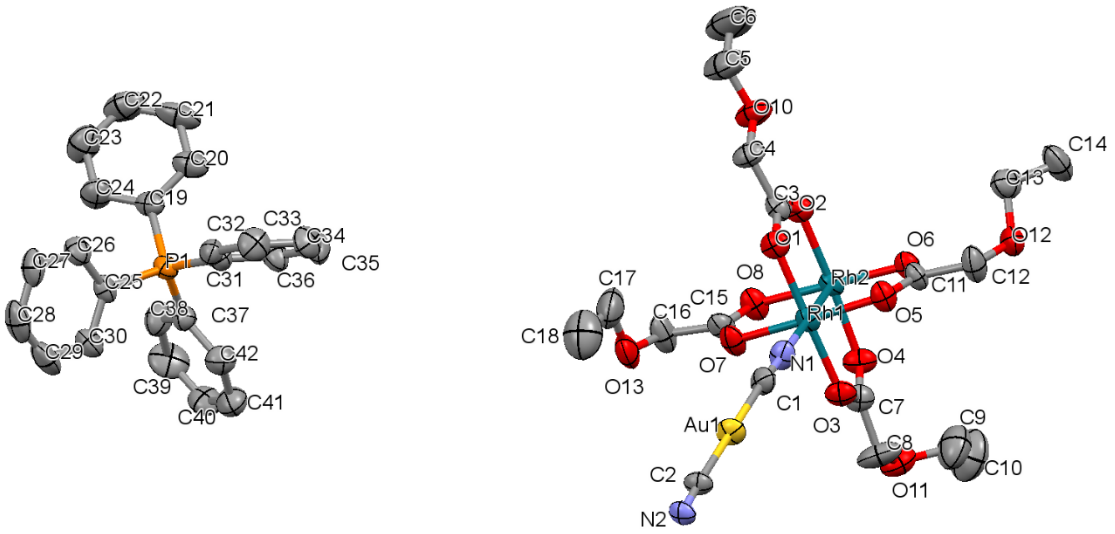

Figure S4. Representation of the asymmetric unit of $(PPh_4)_n[Rh_2(\mu\text{-}O_2CCH_2OEt)_4Au(CN)_2]_n$ (**5**) (50% probability ellipsoids). Rhodium: turquoise; oxygen: red; carbon: grey; nitrogen: purple; gold: yellow; phosphorus: orange. Hydrogen atoms are omitted for clarity.

Table S4. Selected bond lengths [Å] and angles [°] for $(PPh_4)_n[Rh_2(\mu\text{-}O_2CCH_2OEt)_4Au(CN)_2]_n$ (**5**).

	**Bond length(Å)**		**Angle (º)**
Rh(1)-Rh(2)	2.4133(8)	O(1)-Rh(1)-Rh(2)	88.40(13)
O(1)-Rh(1)	2.034(5)	O(3)-Rh(1)-Rh(2)	87.70(14)
O(3)-Rh(1)	2.042(5)	O(5)-Rh(1)-Rh(2)	87.90(13)
O(5)-Rh(1)	2.027(5)	O(7)-Rh(1)-Rh(2)	87.22(14)
O(7)-Rh(1)	2.041(5)	O(2)-Rh(2)-Rh(1)	87.53(13)
N(1)-Rh(1)	2.249(6)	O(4)-Rh(2)-Rh(1)	87.65(14)
O(2)-Rh(2)	2.063(5)	O(6)-Rh(2)-Rh(1)	87.56(13)
O(4)-Rh(2)	2.041(5)	O(8)-Rh(2)-Rh(1)	88.00(14)
O(6)-Rh(2)	2.047(5)	N(2)-Rh(2)-Rh(1)	174.89(14)
O(8)-Rh(2)	2.028(5)	N(1)-Rh(1)-Rh(2)	177.53(16)
N(2)-Rh(2)	2.238(5)	C(1)-N(1)-Rh(1)	164.2(7)
		C(2)#2-N(2)-Rh(1)	163.0(6)

Symmetry transformations used to generate equivalent atoms:

#2 -1+X,1/2-Y,-1/2+Z

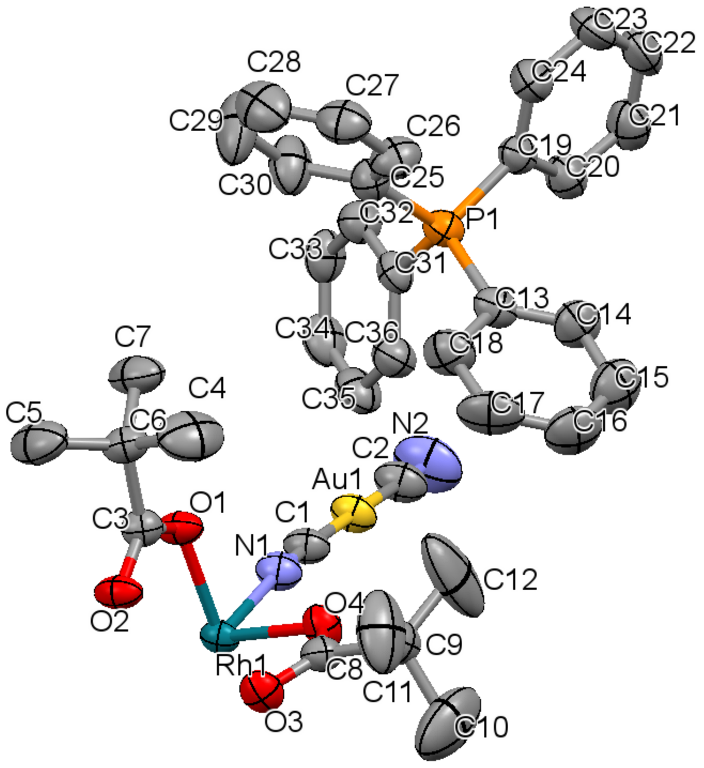

Figure S5. Representation of the asymmetric unit of $(PPh_4)_2\{Rh_2(\mu\text{-}O_2CCMe_3)_4[Au(CN)_2]_2\}$ (**6**) (50% probability ellipsoids). Rhodium: turquoise; oxygen: red; carbon: grey; nitrogen: purple; gold: yellow; phosphorus: orange. Hydrogen atoms are omitted for clarity.

Table S5. Selected bond lengths [Å] and angles [°] for $(PPh_4)_2\{Rh_2(\mu\text{-}O_2CCMe_3)_4[Au(CN)_2]_2\}$ (**6**).

	**Bond length(Å)**		**Angle (º)**
Rh(1)-Rh(1)#1	2.4002(6)	O(4)-Rh(1)-Rh(1)#1	87.88(8)
O(1)-Rh(1)	2.036(2)	O(3)#1-Rh(1)-Rh(1)#1	88.05(8)
O(4)-Rh(1)	2.035(3)	O(1)-Rh(1)-Rh(1)#1	88.24(7)
N(1)-Rh(1)	2.226(4)	O(2)#1-Rh(1)-Rh(1)#1	87.41(7)
		N(1)-Rh(1)-Rh(1)#1	179.24(9)
		C(1)-N(1)-Rh(1)	171.3(3)

Symmetry transformations used to generate equivalent atoms:

#1 -x+2,-y+1,-z+1

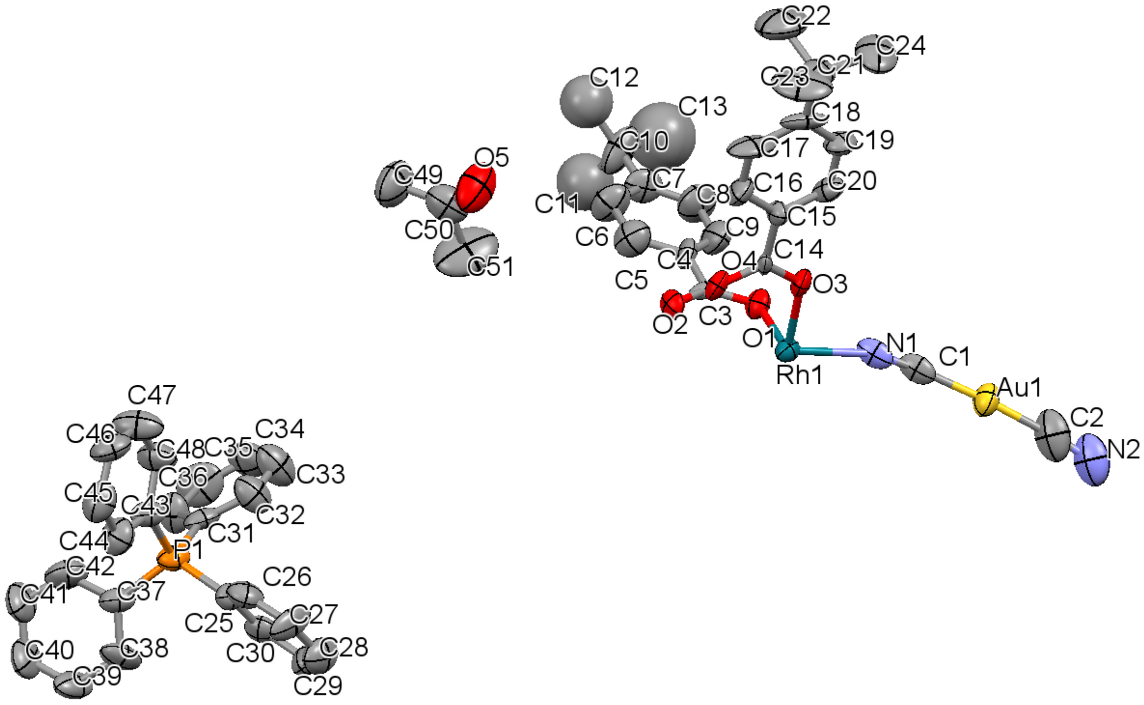

Figure S6. Representation of the asymmetric unit of $(PPh_4)_2\{Rh_2(\mu\text{-}O_2CC_6H_4\text{-}p\text{-}CMe_3)_4[Au(CN)_2]_2\}\cdot 2OCMe_2$ (**7·2OCMe₂**) (50% probability ellipsoids). Rhodium: turquoise; oxygen: red; carbon: grey; nitrogen: purple; gold: yellow; phosphorus: orange. Hydrogen atoms are omitted for clarity.

Table S6. Selected bond lengths [Å] and angles [°] for $(PPh_4)_2\{Rh_2(\mu\text{-}O_2CC_6H_4\text{-}p\text{-}CMe_3)_4[Au(CN)_2]_2\}\cdot 2OCMe_2$ (**7·2OCMe₂**).

	**Bond length(Å)**		**Angle (º)**
Rh(1)-Rh(1)#1	2.3969(19)	O(1)-Rh(1)-Rh(1)#1	88.5(2)
O(1)-Rh(1)	1.989(9)	O(2)#1-Rh(1)-Rh(1)#1	88.2(2)
O(3)-Rh(1)	2.026(8)	O(4)#1-Rh(1)-Rh(1)#1	88.2(2)
N(1)-Rh(1)	2.291(11)	O(3)-Rh(1)-Rh(1)#1	87.1(2)
		N(1)-Rh(1)-Rh(1)#1	176.1(3)
		C(1)-N(1)-Rh(1)	162.4(12)

Symmetry transformations used to generate equivalent atoms:

#1 -x+1,-y+1,-z+2   4

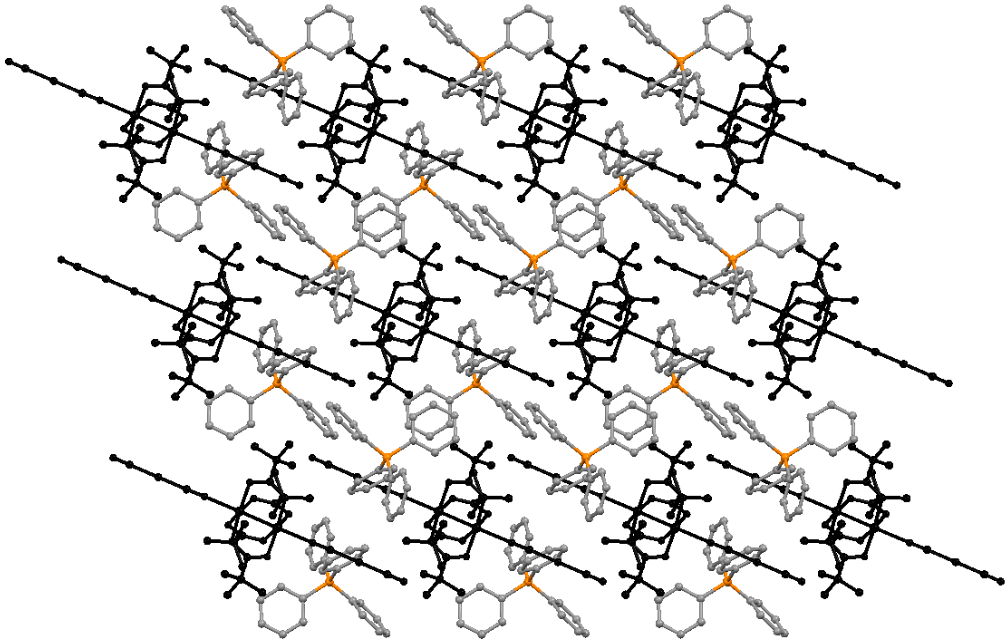

Figure S7. 3x3x3 packing along the *c* axis of the structure of **6**. Discrete dirhodium units are shown in black.

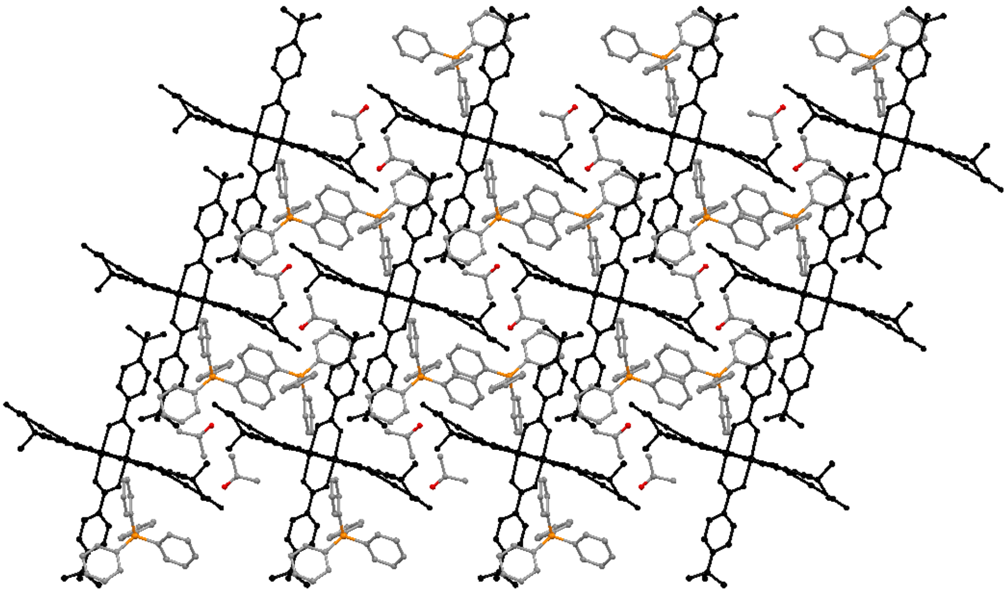

Figure S8. 3x3x3 packing along the *b* axis of the structure of **7·2OCMe$_2$**. Discrete dirhodium units are shown in black.

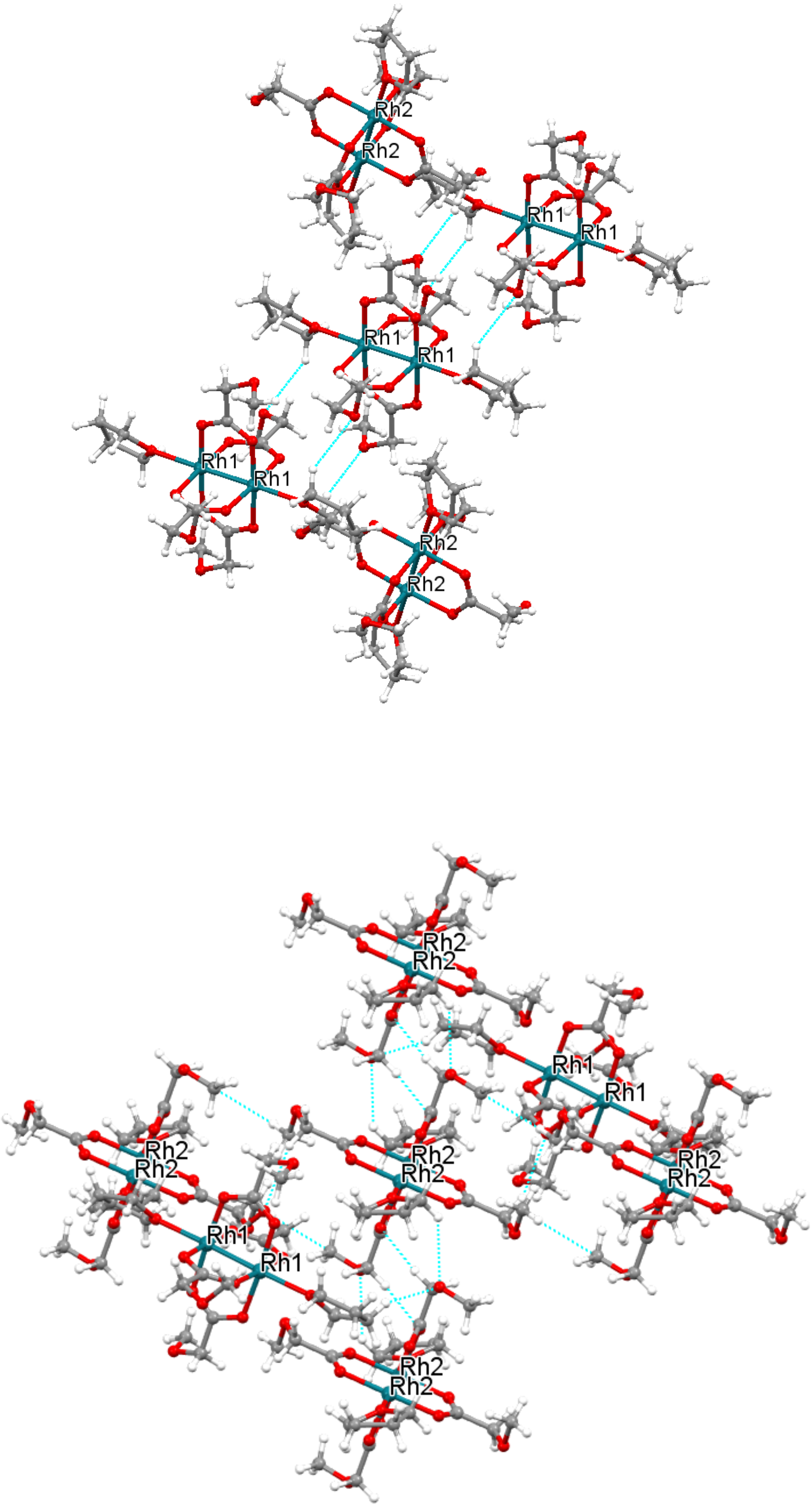

Figure S9. View of the CH···O contacts between Rh1-Rh1 (top) and Rh2-Rh2 (bottom) dirhodium units and neighboring units in the structure of **1**.

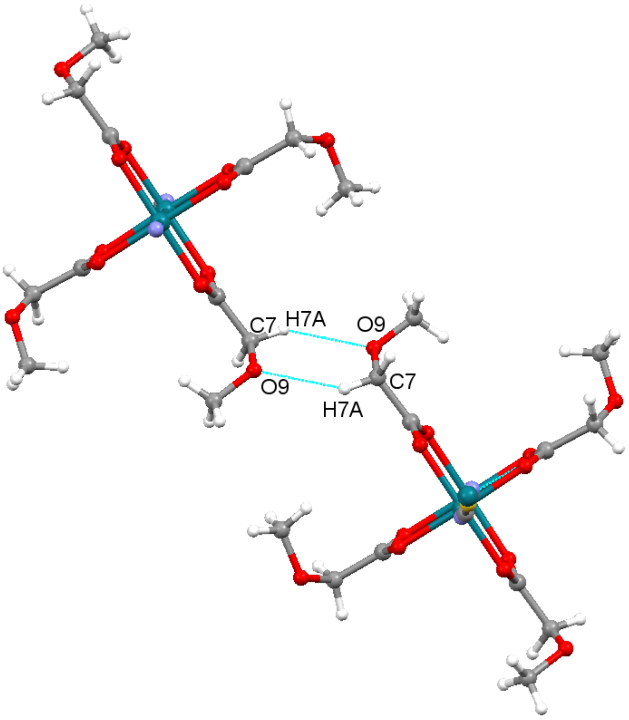

Figure S10. View of the CH···O contacts between neighbor chains in the structure of **4·3CH$_2$Cl$_2$**.

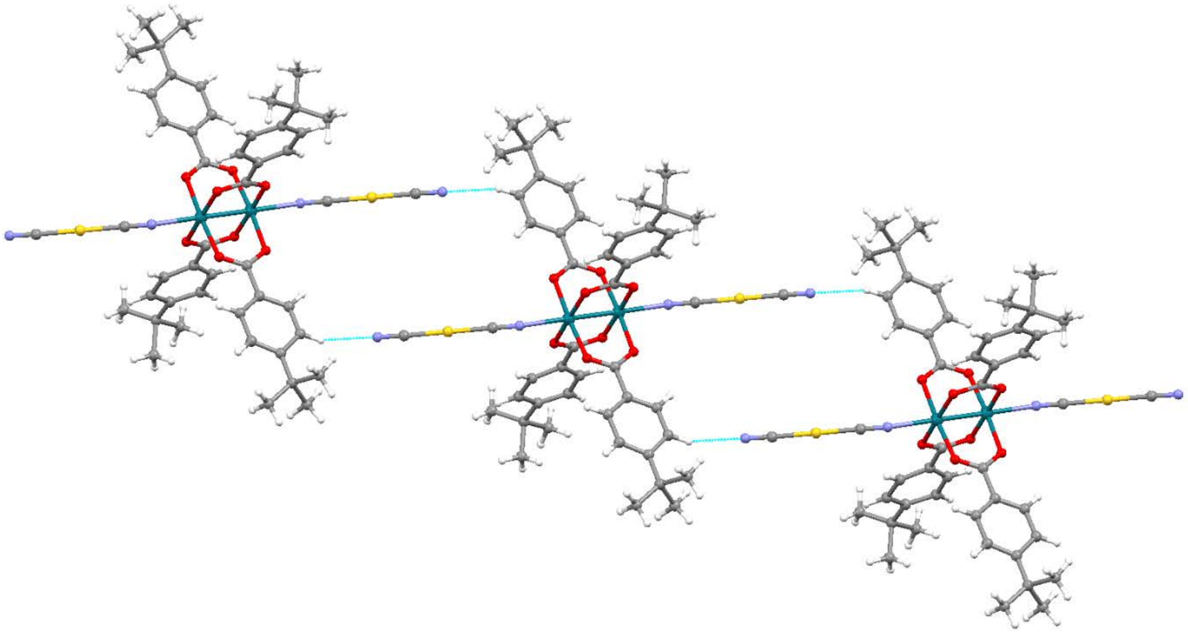

Figure S11. View of the CH···N contacts between neighbor dirhodium units in the structure of **7·2OCMe$_2$**.

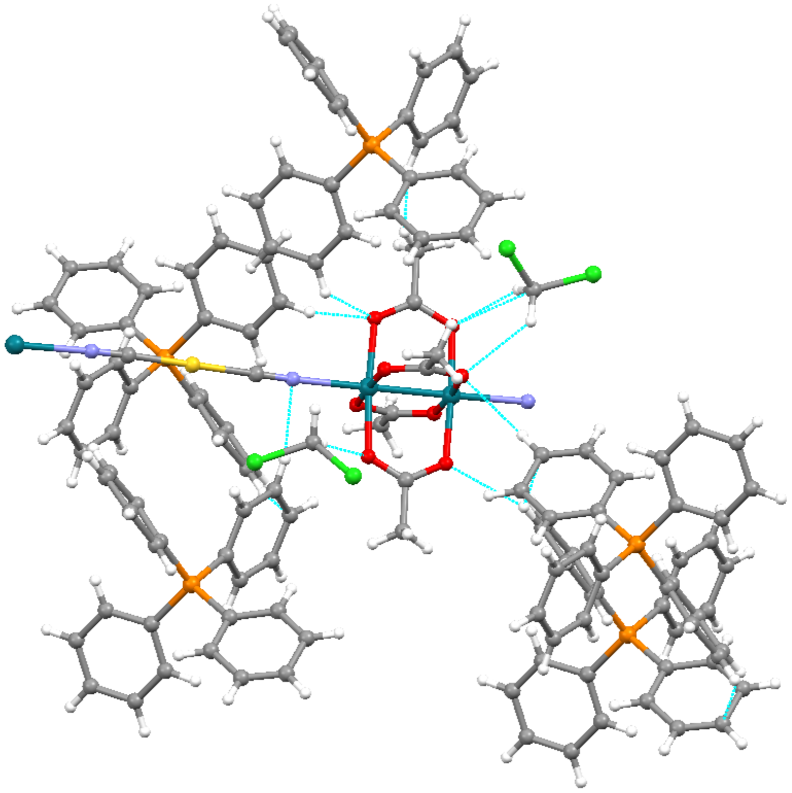

Figure S12. View of the CH···O and CH···N contacts between dirhodium units and tetraphenylphosphonium cations and dichloromethane molecules in the structure of **3·2CH$_2$Cl$_2$**.

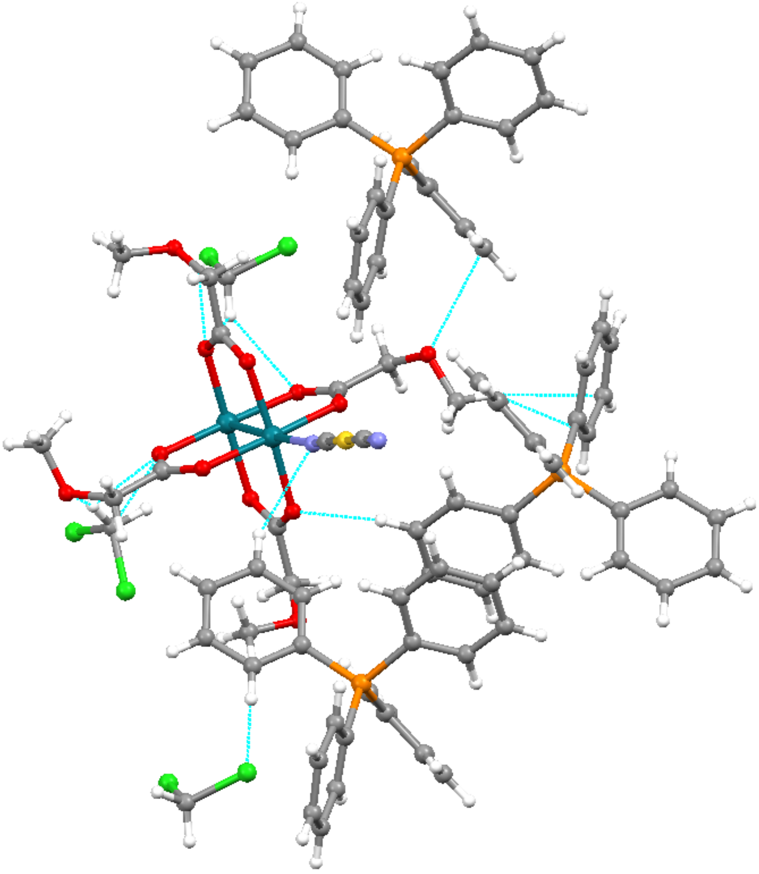

Figure S13. View of the CH···O and CH···N contacts between dirhodium units and tetraphenylphosphonium cations and dichloromethane molecules in the structure of **4·3CH_2Cl_2**.

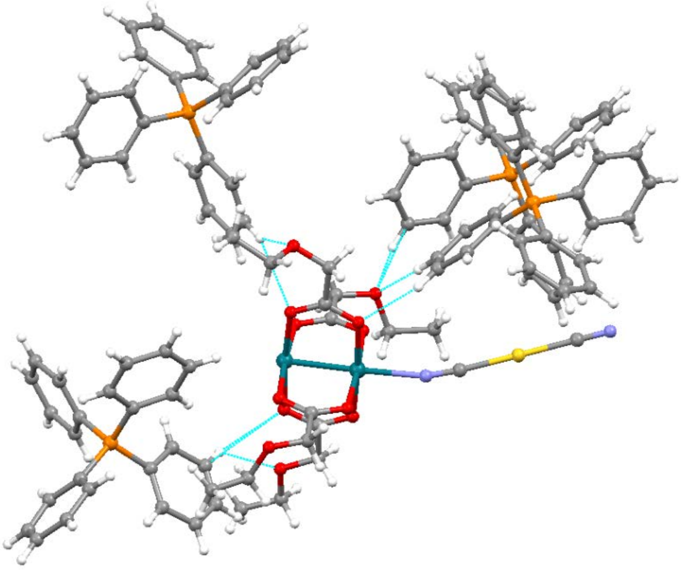

Figure S14. View of the CH···O contacts between dirhodium units and tetraphenylphosphonium cations in the structure of **5**.

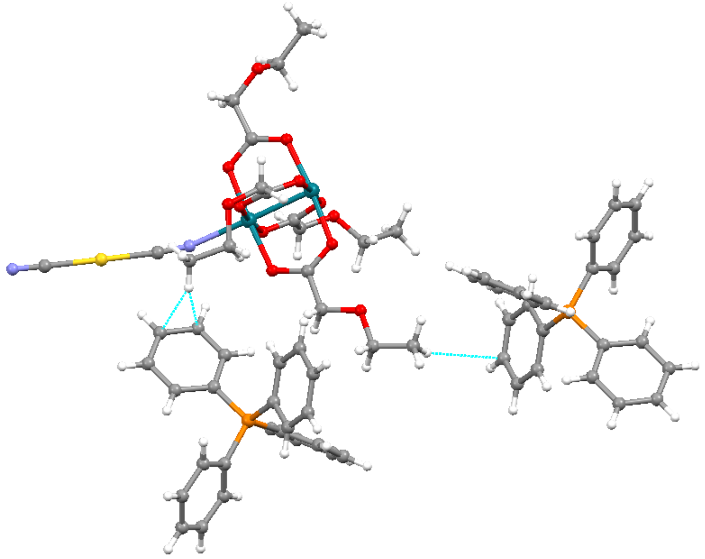

Figure S15. View of the CH···π interactions between dirhodium units and tetraphenylphosphonium cations in the structure of **5**.

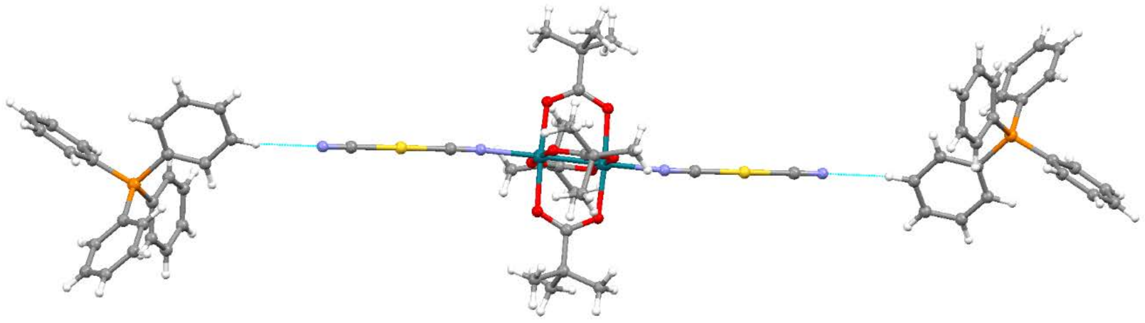

Figure S16. View of the CH···N contacts between dirhodium units and tetraphenylphosphonium cations in the structure of **6**.

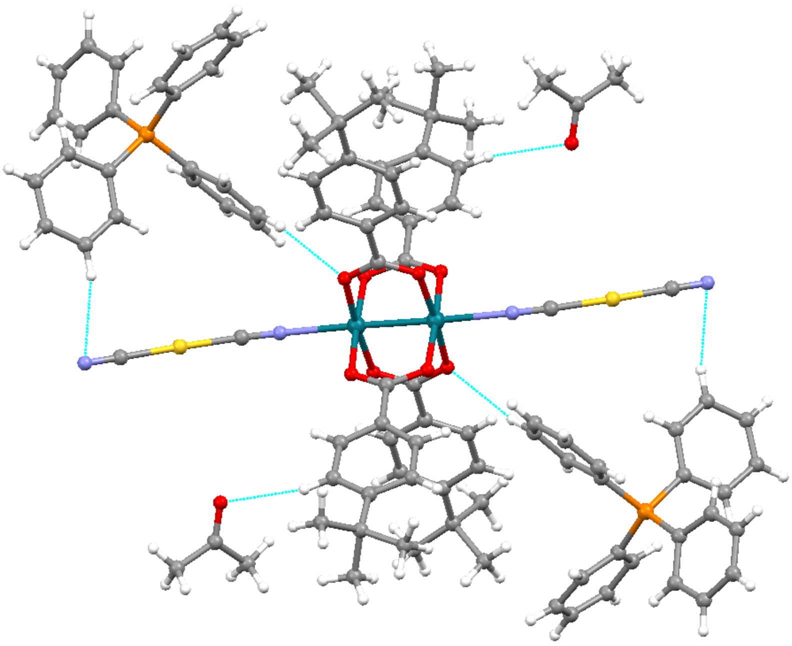

Figure S17. View of the CH···O and CH···N contacts between dirhodium units and tetraphenylphosphonium cations and acetone molecules in the structure of **7·2OCMe₂**.

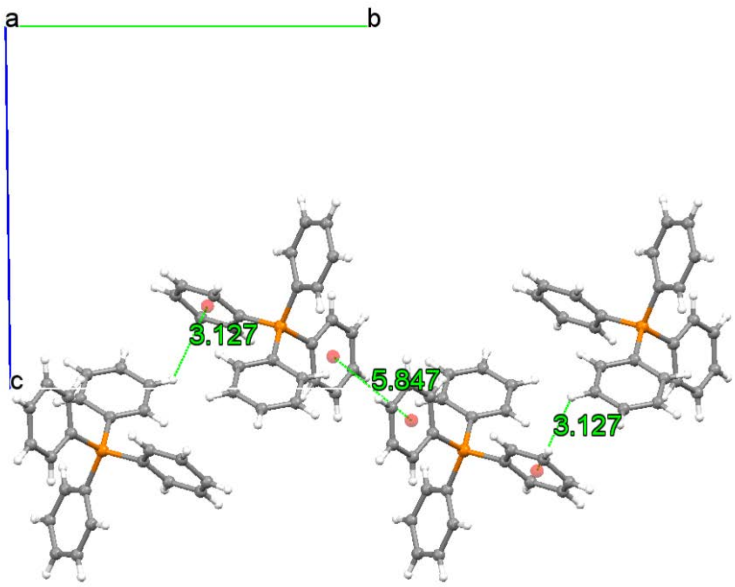

Figure S18. View along the *a* axis of the closest tetraphenylphosphonium cations and the CH···π interactions between them in the structure of of **3·2CH₂Cl₂**. Distances are shown in Å.

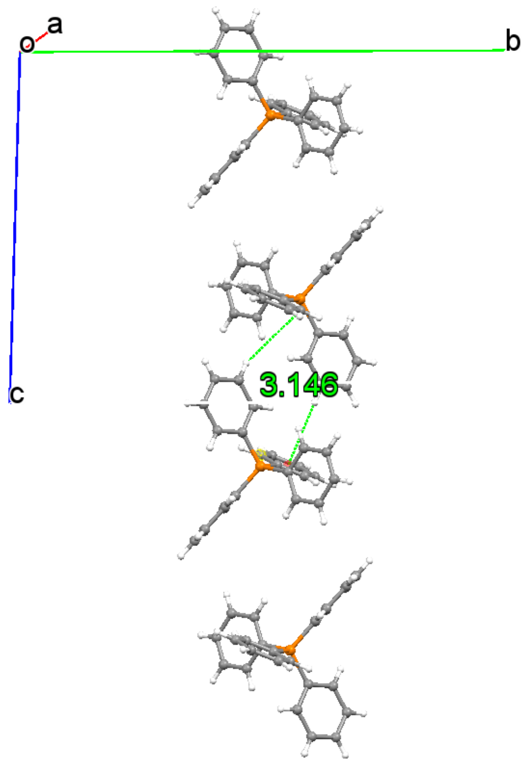

Figure S19. View along the *a* axis of the closest tetraphenylphosphonium cations and the CH···π interactions between them in the structure of **5**. Distances are shown in Å.
